# Crystal structure of 3,4-dimethyl 2-(*tert*-butyl­amino)-5-[2-oxo-4-(thio­morpholin-4-yl)-2*H*-chromen-3-yl]furan-3,4-di­carboxyl­ate ethyl acetate hemisolvate

**DOI:** 10.1107/S2056989015021970

**Published:** 2015-11-28

**Authors:** Tetsuji Moriguchi, Venkataprasad Jalli, Suvratha Krishnamurthy, Akihiko Tsuge, Kenji Yoza

**Affiliations:** a Department of Applied Chemistry, Graduate School of Engineering, Kyushu Institute of Technology, 1-1 Sensui-cho, Tobata-ku, Kitakyushu 804-8550, Japan; b Japan Bruker AXS K.K.3-9, Moriya-cho Kanagawaku Yokohama 221-0022, Japan

**Keywords:** crystal structure, coumarins, thio­morpholine ring, hydrogen bonding

## Abstract

In the title hemisolvate, C_25_H_28_N_2_O_7_S·0.5C_4_H_8_O_2_, the thio­morpholine ring adopts a chair conformation, with the exocyclic N—C bond in an equatorial orientation. The dihedral angle between the coumarin ring system (r.m.s. deviation = 0.044 Å) and the furan ring is 64.84 (6)°. An intra­molecular N—H⋯O hydrogen bond closes an *S*(6) ring. The ethyl acetate solvent mol­ecule is disordered about a crystallographic inversion centre. In the crystal, the components are linked by C—H⋯O and C—H⋯S hydrogen bonds, generating a three-dimensional network.

## Related literature   

For the syntheses and properties of coumarins, see: Arango *et al.* (2010 ▸); Chodankar & Seshadri (1985 ▸); Khan & Kulkarni (1999 ▸); Kitamura *et al.* (2005 ▸); Luo *et al.* (2012 ▸); Sawa *et al.* (2006 ▸); Schiedel *et al.* (2001 ▸); Udaya Kumari *et al.* (2000 ▸); Zen *et al.* (2014 ▸).
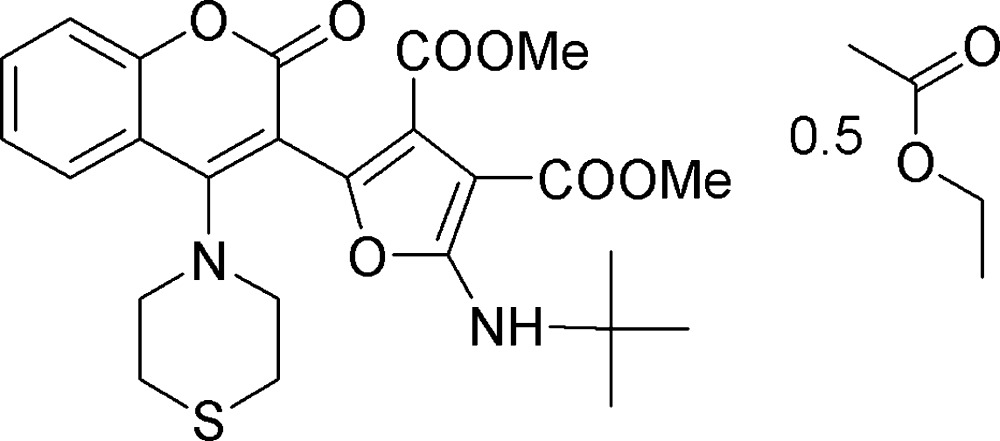


## Experimental   

### Crystal data  

C_25_H_28_N_2_O_7_S·0.5C_4_H_8_O_2_
*M* *_r_* = 544.61
Monoclinic, 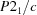

*a* = 14.3733 (17) Å
*b* = 16.1159 (19) Å
*c* = 11.7019 (14) Å
β = 95.007 (1)°
*V* = 2700.3 (6) Å^3^
*Z* = 4
Mo *K*α radiation
μ = 0.17 mm^−1^
*T* = 90 K
0.50 × 0.40 × 0.25 mm

### Data collection  

Bruker APEXII diffractometer
Absorption correction: multi-scan (*SADABS*; Bruker, 2009 ▸) *T*_min_ = 0.853, *T*_max_ = 0.958
25410 measured reflections
4757 independent reflections
4316 reflections with *I* > 2σ(*I*)
*R*_int_ = 0.022

### Refinement  

*R*[*F* ^2^ > 2σ(*F* ^2^)] = 0.032
*wR*(*F* ^2^) = 0.086
*S* = 1.02
4757 reflections
375 parameters
30 restraints
H-atom parameters constrained
Δρ_max_ = 0.26 e Å^−3^
Δρ_min_ = −0.23 e Å^−3^

### 

Data collection: *APEX2* (Bruker, 2009 ▸); cell refinement: *SAINT* (Bruker, 2009 ▸); data reduction: *SAINT*; program(s) used to solve structure: *SHELXS97* (Sheldrick, 2008 ▸); program(s) used to refine structure: *SHELXL97* (Sheldrick, 2008 ▸); molecular graphics: *Mercury* (Macrae *et al.*, 2008 ▸); software used to prepare material for publication: *SHELXL97*.

## Supplementary Material

Crystal structure: contains datablock(s) global, I. DOI: 10.1107/S2056989015021970/hb7531sup1.cif

Structure factors: contains datablock(s) I. DOI: 10.1107/S2056989015021970/hb7531Isup2.hkl

Supporting information file. DOI: 10.1107/S2056989015021970/hb7531Isup3.cml

**Fig. 1.**
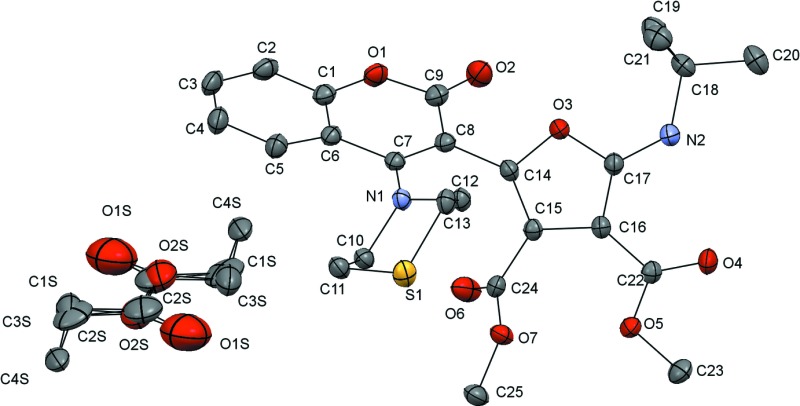
Molecular configuration and atom-numbering scheme for the title compound with displacement ellipsoids drawn at the 50% probability level. Hydrogen atoms are omitted for clarity.

. DOI: 10.1107/S2056989015021970/hb7531fig1.tif
Mol­ecular configuration and atom-numbering scheme for the title compound with displacement ellipsoids drawn at the 50% probability level. Hydrogen atoms are omitted for clarity.

**Fig. 2.**
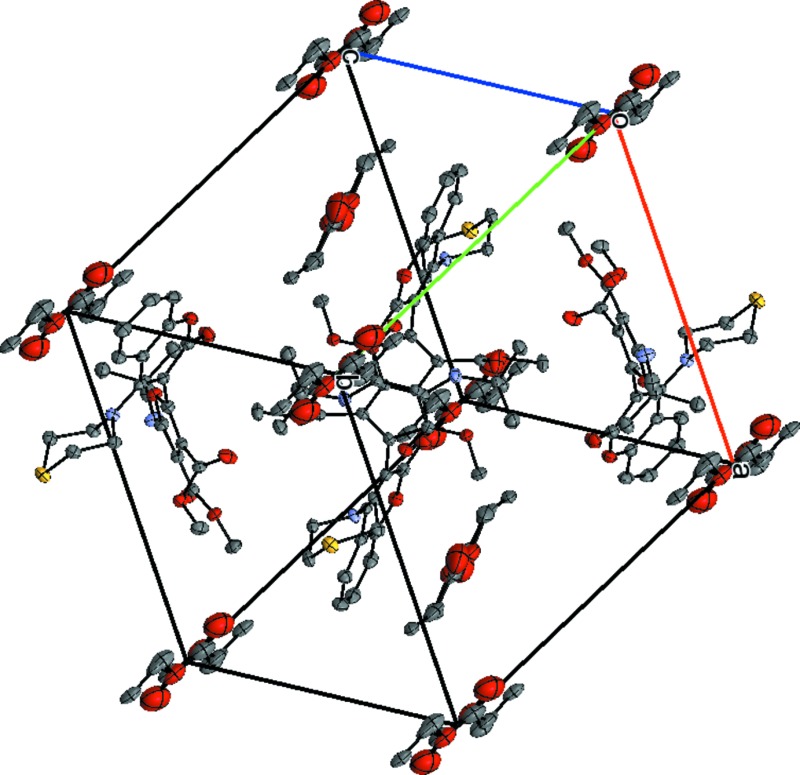
Crystal packing diagram of the title compound.

. DOI: 10.1107/S2056989015021970/hb7531fig2.tif
Crystal packing diagram of the title compound.

**Fig. 3.**
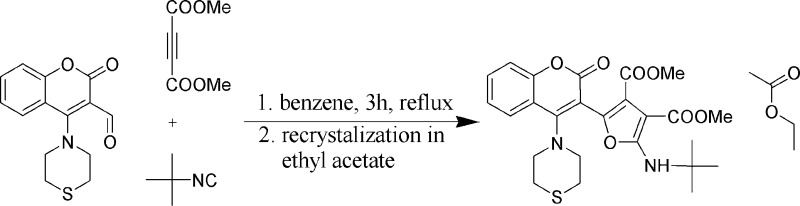
Chemical scheme of title compound with solvent molecule. In the cystal system the main molecule and solvent molecule was found in 1:0.5 ratio.

. DOI: 10.1107/S2056989015021970/hb7531fig3.tif
Chemical scheme of title compound with solvent mol­ecule. In the cystal system the main mol­ecule and solvent mol­ecule was found in 1:0.5 ratio.

**Fig. 4.**
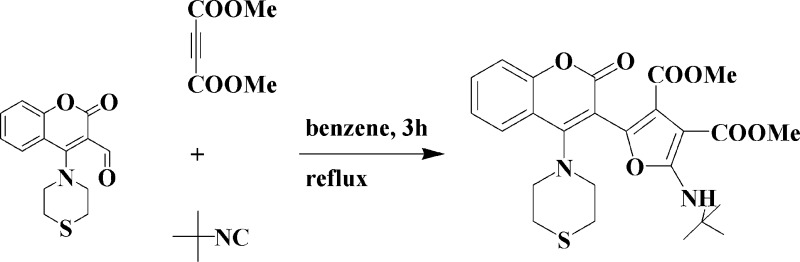
Synthesis of title compound (I).

. DOI: 10.1107/S2056989015021970/hb7531fig4.tif
Synthesis of title compound (I).

CCDC reference: 1432824

Additional supporting information:  crystallographic information; 3D view; checkCIF report

## Figures and Tables

**Table 1:** Hydrogen-bond geometry (Å, °)

*D*—H⋯*A*	*D*—H	H⋯*A*	*D*⋯*A*	*D*—H⋯*A*
N2—H13⋯O4	0.86	2.25	2.8255 (17)	125
C3—H2⋯O6^i^	0.93	2.41	3.325 (2)	167
C12—H9⋯O4^ii^	0.97	2.44	3.1531 (18)	130
C12—H10⋯O1*S* ^iii^	0.97	2.48	3.208 (5)	132
C19—H15⋯O3	0.96	2.42	3.0090 (19)	119
C23—H23⋯S1^iv^	0.96	2.78	3.5639 (17)	139
C25—H26⋯S1^v^	0.96	2.83	3.7406 (18)	159

Symmetry codes: (i) 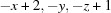; (ii) 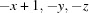; (iii) 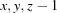; (iv) 
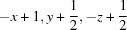
; (v) 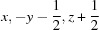.

## supplementary crystallographic information

### S1. Structural commentary

Coumarins analogs having furan heterocycle have gained significant importance because of their properties as anti-leishmania panamensis, dyes and fluorescent sensors. For the activity related reports of furyl coumarins, see: Arango *et al.* (2010); Zen *et al.* (2014); Schiedel *et al.* (2001); Kitamura *et al.* (2005). Natural furyl coumarin derivatives extracted from plants such as microminutin, micromelin, psoralen, 8-meth­oxy­psoralen have important properties in medicinal chemistry and bio photochemistry. For the activity related reports of natural furyl coumarins, see: Luo *et al.* (2012). It was well documented that by introducing a heteroaromatic substituent at 3-position the absorption and emission maxima of coumarin scaffold can be improved because of extended π conjugation and consequently their optoelectronic properties can be improved. Due to their versatile properties a variety of 3-hetero­aryl coumarin derivatives have been synthesized and tested for their optoelectronic properties. For the optoelectronic properties of coumarin derivatives, see: Sawa *et al.* (2006). For the synthesis related reports of 3-furyl coumarin derivatives, see: Chodankar *et al.* (1985); Khan & Kulkarni (1999); Udaya Kumari *et al.* (2000). Thus, the elucidation of the crystal structures of coumarin derivatives has attracted much attention. Here,we report the crystal structure of the title compound, (I).

### S2. Synthesis and crystallization,

A solution of 4-thio­morpholino-3-formyl coumarin (1 mmol), dmethyl acetyl­enedi­carboxyl­ate (1 mmol), t-butyl isocyanide (1 mmol) were refluxed at 80°C for 3h. The volatiles were removed under reduced pressure. The crude reaction mixture was subjected to column chromatography using EtOAc/Hexane mobile phase. The title compound was isolated as yellow color solid with 80% yield. Yellow prisms were obtained by vapour diffusion method at room temperature, i.e., hexane vapour was allowed to diffuse into an EtOAc solution of 4-thio­morpholino-3-(2-N-t-butyl­amino-3,4-di­methyl­carboxyl­ate-5-furyl) 2H-1-benzo­pyran-2-one at room temperature.

mp 107-109 °C; IR; ν_max_(KBr) 3288, 1732, 1728, 1667, 1658, 1618, 1418, 1240, 1041 cm^-1^; *δ*_H_ (500 MHz CDCl_3_) 7.69 (1 H, d), 7.52 (1 H, t), 7.28-7.33 (2 H, dd), 7.05 (1 H, s), 3.78 (3 H, s), 3.75 (3 H, s), 3.39-3.48 (4 H, m), 2.77-2.81 (4 H, m), 1.44 (9 H, s); *δ*_C_ (125 MHz, CDCl_3_) 165.6, 163.9, 163.0, 161.2, 160.6, 153.4, 138.2, 132.4, 125.1, 123.7, 118.7, 118.1, 117.7, 103.6, 87.8, 53.2, 52.7, 51.8, 51.3, 28.0; LCMS: MH^+^, 501.

### Crystal data

**Table:**

C_25_H_28_N_2_O_7_S·0.5C_4_H_8_O_2_	*F*(000) = 1152
*M**_r_* = 544.61	*D*_x_ = 1.340 Mg m^−^^3^
Monoclinic, *P*2_1_/*c*	Mo *K*α radiation, λ = 0.71073 Å
Hall symbol: -P 2ybc	Cell parameters from 25410 reflections
*a* = 14.3733 (17) Å	θ = 1.4–25.0°
*b* = 16.1159 (19) Å	µ = 0.17 mm^−^^1^
*c* = 11.7019 (14) Å	*T* = 90 K
β = 95.007 (1)°	Prism, yellow
*V* = 2700.3 (6) Å^3^	0.50 × 0.40 × 0.25 mm
*Z* = 4	

### Data collection

**Table:**

Bruker APEXII diffractometer	4757 independent reflections
Radiation source: fine-focus sealed tube	4316 reflections with *I* > 2σ(*I*)
Graphite monochromator	*R*_int_ = 0.022
Detector resolution: 8.333 pixels mm^-1^	θ_max_ = 25.0°, θ_min_ = 1.4°
ω scans	*h* = −17→17
Absorption correction: multi-scan (*SADABS*; Bruker, 2009)	*k* = −19→19
*T*_min_ = 0.853, *T*_max_ = 0.958	*l* = −13→13
25410 measured reflections	

### Refinement

**Table:**

Refinement on *F*^2^	Primary atom site location: structure-invariant direct methods
Least-squares matrix: full	Secondary atom site location: difference Fourier map
*R*[*F*^2^ > 2σ(*F*^2^)] = 0.032	Hydrogen site location: inferred from neighbouring sites
*wR*(*F*^2^) = 0.086	H-atom parameters constrained
*S* = 1.02	*w* = 1/[σ^2^(*F*_o_^2^) + (0.040*P*)^2^ + 1.5222*P*] where *P* = (*F*_o_^2^ + 2*F*_c_^2^)/3
4757 reflections	(Δ/σ)_max_ < 0.001
375 parameters	Δρ_max_ = 0.26 e Å^−^^3^
30 restraints	Δρ_min_ = −0.23 e Å^−^^3^

### Special details

**Table:**

Geometry. All e.s.d.'s (except the e.s.d. in the dihedral angle between two l.s. planes) are estimated using the full covariance matrix. The cell e.s.d.'s are taken into account individually in the estimation of e.s.d.'s in distances, angles and torsion angles; correlations between e.s.d.'s in cell parameters are only used when they are defined by crystal symmetry. An approximate (isotropic) treatment of cell e.s.d.'s is used for estimating e.s.d.'s involving l.s. planes.
Refinement. Refinement of *F*^2^ against ALL reflections. The weighted *R*-factor *wR* and goodness of fit *S* are based on *F*^2^, conventional *R*-factors *R* are based on *F*, with *F* set to zero for negative *F*^2^. The threshold expression of *F*^2^ > σ(*F*^2^) is used only for calculating *R*-factors(gt) *etc*. and is not relevant to the choice of reflections for refinement. *R*-factors based on *F*^2^ are statistically about twice as large as those based on *F*, and *R*- factors based on ALL data will be even larger.

### Fractional atomic coordinates and isotropic or equivalent isotropic displacement parameters (Å^2^)

**Table:**

	*x*	*y*	*z*	*U*_iso_*/*U*_eq_	Occ. (<1)
C1	0.99995 (10)	0.03172 (9)	0.34623 (12)	0.0231 (3)	
C2	1.08898 (11)	0.03216 (10)	0.40315 (13)	0.0282 (3)	
H1	1.1151	0.0815	0.4321	0.034*	
C3	1.13819 (11)	−0.04082 (11)	0.41634 (14)	0.0316 (4)	
H2	1.197	−0.0413	0.4563	0.038*	
C4	1.09998 (11)	−0.11405 (11)	0.36986 (14)	0.0310 (4)	
H3	1.1343	−0.163	0.3759	0.037*	
C5	1.01118 (10)	−0.11389 (10)	0.31490 (13)	0.0257 (3)	
H4	0.9864	−0.1631	0.2837	0.031*	
C6	0.95710 (10)	−0.04105 (9)	0.30487 (12)	0.0208 (3)	
C7	0.86234 (9)	−0.03596 (9)	0.24765 (11)	0.0190 (3)	
C8	0.82094 (10)	0.04097 (9)	0.23612 (12)	0.0199 (3)	
C9	0.87117 (10)	0.11639 (9)	0.27233 (13)	0.0239 (3)	
C10	0.81001 (10)	−0.18053 (9)	0.27629 (12)	0.0215 (3)	
H5	0.7459	−0.1825	0.2962	0.026*	
H6	0.8502	−0.1745	0.3468	0.026*	
C11	0.83315 (11)	−0.26140 (9)	0.21796 (12)	0.0242 (3)	
H7	0.8264	−0.3073	0.2703	0.029*	
H8	0.8976	−0.2601	0.1993	0.029*	
C12	0.77517 (10)	−0.17887 (9)	0.02143 (12)	0.0238 (3)	
H10	0.8391	−0.1757	0.0011	0.029*	
H9	0.7342	−0.1748	−0.0488	0.029*	
C13	0.75641 (10)	−0.10617 (9)	0.09892 (12)	0.0217 (3)	
H11	0.7635	−0.0545	0.0581	0.026*	
H12	0.6928	−0.1092	0.1205	0.026*	
C14	0.72271 (10)	0.05441 (8)	0.19528 (12)	0.0201 (3)	
C15	0.64058 (9)	0.03168 (8)	0.23338 (12)	0.0185 (3)	
C16	0.56676 (10)	0.06269 (9)	0.15304 (12)	0.0194 (3)	
C17	0.61162 (10)	0.10511 (9)	0.07106 (12)	0.0214 (3)	
C18	0.62518 (11)	0.20817 (9)	−0.09030 (12)	0.0243 (3)	
C19	0.68655 (12)	0.26826 (10)	−0.01648 (14)	0.0323 (4)	
H14	0.6495	0.296	0.0363	0.048*	
H16	0.7128	0.3086	−0.0648	0.048*	
H15	0.736	0.2381	0.0255	0.048*	
C20	0.54811 (12)	0.25574 (10)	−0.16032 (14)	0.0320 (4)	
H17	0.5063	0.2172	−0.2007	0.048*	
H18	0.5754	0.2911	−0.2144	0.048*	
H19	0.5142	0.2889	−0.1099	0.048*	
C21	0.68273 (12)	0.15921 (10)	−0.17061 (14)	0.0323 (4)	
H20	0.727	0.1249	−0.1262	0.048*	
H22	0.7154	0.197	−0.2163	0.048*	
H21	0.642	0.1249	−0.2198	0.048*	
C22	0.46747 (10)	0.06935 (9)	0.16008 (12)	0.0204 (3)	
C23	0.34095 (10)	0.05062 (10)	0.27252 (14)	0.0297 (4)	
H23	0.3276	0.1089	0.2747	0.045*	
H24	0.3263	0.0253	0.343	0.045*	
H25	0.3039	0.0256	0.2095	0.045*	
C24	0.62849 (9)	−0.01445 (9)	0.34073 (12)	0.0200 (3)	
C25	0.55129 (12)	−0.12568 (10)	0.42109 (13)	0.0298 (4)	
H26	0.605	−0.1357	0.4739	0.045*	
H27	0.522	−0.1775	0.3994	0.045*	
H28	0.5078	−0.0912	0.457	0.045*	
C1S	1.0683 (9)	−0.1031 (8)	1.0236 (11)	0.059 (3)	0.5
H1SA	1.1073	−0.0784	1.0852	0.088*	0.5
H1SB	1.1057	−0.1181	0.9628	0.088*	0.5
H1SC	1.0388	−0.1517	1.0511	0.088*	0.5
C2S	0.994 (2)	−0.041 (2)	0.979 (2)	0.0624 (17)	0.5
C3S	0.9217 (9)	0.0898 (7)	0.9635 (11)	0.0506 (17)	0.5
H3SA	0.8679	0.0739	1.0031	0.061*	0.5
H3SB	0.9073	0.0815	0.8817	0.061*	0.5
C4S	0.9519 (2)	0.1897 (2)	0.9924 (3)	0.0399 (9)	0.5
H4SA	0.9024	0.2257	0.9627	0.06*	0.5
H4SB	1.0079	0.2028	0.9569	0.06*	0.5
H4SC	0.9627	0.1971	1.0738	0.06*	0.5
N1	0.82204 (8)	−0.10814 (7)	0.20229 (10)	0.0197 (3)	
N2	0.57584 (9)	0.15043 (8)	−0.01773 (11)	0.0286 (3)	
H13	0.5167	0.1455	−0.0349	0.034*	
O1	0.95818 (7)	0.10811 (6)	0.32980 (9)	0.0263 (2)	
O2	0.84156 (8)	0.18618 (7)	0.25827 (10)	0.0324 (3)	
O3	0.70539 (7)	0.09935 (6)	0.09266 (8)	0.0223 (2)	
O4	0.41429 (7)	0.10217 (7)	0.08665 (9)	0.0299 (3)	
O5	0.43880 (7)	0.03856 (6)	0.25799 (8)	0.0226 (2)	
O6	0.65724 (8)	0.00853 (8)	0.43480 (9)	0.0370 (3)	
O7	0.57991 (7)	−0.08439 (6)	0.32009 (8)	0.0235 (2)	
O1S	0.9264 (3)	−0.0628 (3)	0.9137 (4)	0.1025 (13)	0.5
O2S	1.0034 (13)	0.0428 (14)	1.0052 (12)	0.0550 (15)	0.5
S1	0.75716 (3)	−0.27772 (2)	0.08816 (3)	0.02791 (11)	

### Atomic displacement parameters (Å^2^)

**Table:**

	*U*^11^	*U*^22^	*U*^33^	*U*^12^	*U*^13^	*U*^23^
C1	0.0205 (7)	0.0277 (8)	0.0213 (7)	−0.0025 (6)	0.0037 (6)	0.0008 (6)
C2	0.0218 (8)	0.0373 (9)	0.0252 (8)	−0.0098 (7)	−0.0001 (6)	−0.0009 (7)
C3	0.0166 (7)	0.0500 (10)	0.0272 (8)	−0.0029 (7)	−0.0034 (6)	0.0055 (7)
C4	0.0216 (8)	0.0390 (9)	0.0317 (9)	0.0058 (7)	−0.0020 (6)	0.0040 (7)
C5	0.0219 (7)	0.0290 (8)	0.0256 (8)	0.0008 (6)	−0.0010 (6)	0.0000 (6)
C6	0.0177 (7)	0.0277 (8)	0.0171 (7)	−0.0014 (6)	0.0015 (5)	0.0016 (6)
C7	0.0177 (7)	0.0236 (7)	0.0159 (7)	−0.0013 (6)	0.0026 (5)	0.0023 (5)
C8	0.0178 (7)	0.0231 (7)	0.0191 (7)	−0.0012 (6)	0.0031 (5)	0.0015 (6)
C9	0.0219 (7)	0.0257 (8)	0.0245 (8)	−0.0017 (6)	0.0043 (6)	0.0002 (6)
C10	0.0219 (7)	0.0227 (7)	0.0194 (7)	−0.0020 (6)	−0.0001 (6)	0.0041 (6)
C11	0.0270 (8)	0.0221 (7)	0.0229 (7)	−0.0015 (6)	−0.0020 (6)	0.0043 (6)
C12	0.0228 (7)	0.0283 (8)	0.0194 (7)	0.0022 (6)	−0.0035 (6)	0.0008 (6)
C13	0.0210 (7)	0.0228 (7)	0.0202 (7)	0.0006 (6)	−0.0046 (6)	0.0018 (6)
C14	0.0224 (7)	0.0175 (7)	0.0200 (7)	0.0018 (6)	0.0002 (6)	0.0034 (5)
C15	0.0191 (7)	0.0168 (7)	0.0194 (7)	0.0016 (5)	0.0004 (5)	−0.0004 (5)
C16	0.0189 (7)	0.0200 (7)	0.0187 (7)	0.0018 (5)	−0.0008 (5)	0.0010 (5)
C17	0.0195 (7)	0.0226 (7)	0.0218 (7)	0.0028 (6)	−0.0004 (6)	0.0019 (6)
C18	0.0309 (8)	0.0213 (7)	0.0209 (7)	0.0007 (6)	0.0030 (6)	0.0039 (6)
C19	0.0402 (9)	0.0246 (8)	0.0314 (9)	−0.0006 (7)	−0.0010 (7)	0.0005 (7)
C20	0.0425 (10)	0.0277 (8)	0.0251 (8)	0.0061 (7)	−0.0007 (7)	0.0049 (6)
C21	0.0378 (9)	0.0299 (8)	0.0300 (8)	0.0010 (7)	0.0075 (7)	−0.0007 (7)
C22	0.0203 (7)	0.0194 (7)	0.0209 (7)	0.0005 (6)	−0.0003 (6)	−0.0014 (6)
C23	0.0207 (8)	0.0340 (9)	0.0353 (9)	0.0037 (6)	0.0082 (6)	0.0009 (7)
C24	0.0148 (6)	0.0229 (7)	0.0219 (7)	0.0010 (5)	−0.0006 (5)	0.0021 (6)
C25	0.0328 (9)	0.0313 (8)	0.0249 (8)	−0.0065 (7)	0.0008 (6)	0.0099 (7)
C1S	0.036 (4)	0.090 (5)	0.055 (4)	−0.004 (3)	0.028 (3)	−0.002 (3)
C2S	0.049 (3)	0.082 (3)	0.060 (4)	−0.003 (2)	0.026 (3)	0.013 (3)
C3S	0.036 (3)	0.070 (3)	0.047 (3)	0.013 (3)	0.007 (2)	0.020 (3)
C4S	0.0258 (17)	0.052 (2)	0.044 (2)	0.0120 (15)	0.0153 (15)	0.0192 (17)
N1	0.0199 (6)	0.0199 (6)	0.0185 (6)	−0.0012 (5)	−0.0039 (5)	0.0028 (5)
N2	0.0200 (6)	0.0372 (8)	0.0280 (7)	0.0004 (5)	−0.0007 (5)	0.0136 (6)
O1	0.0223 (5)	0.0244 (5)	0.0319 (6)	−0.0049 (4)	0.0000 (4)	−0.0021 (4)
O2	0.0326 (6)	0.0207 (6)	0.0437 (7)	0.0006 (5)	0.0019 (5)	−0.0024 (5)
O3	0.0185 (5)	0.0251 (5)	0.0232 (5)	0.0016 (4)	0.0023 (4)	0.0073 (4)
O4	0.0204 (5)	0.0408 (7)	0.0277 (6)	0.0047 (5)	−0.0027 (4)	0.0086 (5)
O5	0.0175 (5)	0.0271 (5)	0.0236 (5)	0.0021 (4)	0.0035 (4)	0.0028 (4)
O6	0.0417 (7)	0.0463 (7)	0.0214 (6)	−0.0190 (6)	−0.0069 (5)	0.0048 (5)
O7	0.0284 (5)	0.0218 (5)	0.0202 (5)	−0.0045 (4)	0.0014 (4)	0.0039 (4)
O1S	0.104 (3)	0.113 (3)	0.094 (3)	−0.019 (3)	0.029 (2)	0.021 (3)
O2S	0.0419 (18)	0.0739 (19)	0.052 (4)	0.0088 (17)	0.017 (3)	0.014 (3)
S1	0.0304 (2)	0.0225 (2)	0.0294 (2)	−0.00151 (15)	−0.00565 (16)	−0.00288 (15)

### Geometric parameters (Å, º)

**Table:**

C1—O1	1.3759 (18)	C18—N2	1.4817 (19)
C1—C2	1.390 (2)	C18—C21	1.525 (2)
C1—C6	1.392 (2)	C18—C20	1.526 (2)
C2—C3	1.374 (2)	C18—C19	1.526 (2)
C2—H1	0.93	C19—H14	0.96
C3—C4	1.392 (2)	C19—H16	0.96
C3—H2	0.93	C19—H15	0.96
C4—C5	1.378 (2)	C20—H17	0.96
C4—H3	0.93	C20—H18	0.96
C5—C6	1.407 (2)	C20—H19	0.96
C5—H4	0.93	C21—H20	0.96
C6—C7	1.4667 (19)	C21—H22	0.96
C7—C8	1.377 (2)	C21—H21	0.96
C7—N1	1.3844 (18)	C22—O4	1.2197 (17)
C8—C9	1.458 (2)	C22—O5	1.3457 (17)
C8—C14	1.467 (2)	C23—O5	1.4444 (17)
C9—O2	1.2088 (19)	C23—H23	0.96
C9—O1	1.3737 (18)	C23—H24	0.96
C10—N1	1.4721 (18)	C23—H25	0.96
C10—C11	1.521 (2)	C24—O6	1.2001 (18)
C10—H5	0.97	C24—O7	1.3367 (17)
C10—H6	0.97	C25—O7	1.4469 (18)
C11—S1	1.8110 (15)	C25—H26	0.96
C11—H7	0.97	C25—H27	0.96
C11—H8	0.97	C25—H28	0.96
C12—C13	1.520 (2)	C1S—C2S	1.51 (3)
C12—S1	1.8027 (15)	C1S—H1SA	0.96
C12—H10	0.97	C1S—H1SB	0.96
C12—H9	0.97	C1S—H1SC	0.96
C13—N1	1.4680 (17)	C2S—O1S	1.23 (3)
C13—H11	0.97	C2S—O2S	1.396 (16)
C13—H12	0.97	C3S—O2S	1.446 (19)
C14—C15	1.348 (2)	C3S—C4S	1.693 (13)
C14—O3	1.4058 (17)	C3S—H3SA	0.97
C15—C16	1.4440 (19)	C3S—H3SB	0.97
C15—C24	1.4830 (19)	C4S—H4SA	0.96
C16—C17	1.383 (2)	C4S—H4SB	0.96
C16—C22	1.441 (2)	C4S—H4SC	0.96
C17—N2	1.3357 (19)	N2—H13	0.86
C17—O3	1.3527 (17)		
			
O1—C1—C2	115.75 (13)	C18—C19—H14	109.5
O1—C1—C6	122.08 (13)	C18—C19—H16	109.5
C2—C1—C6	122.13 (14)	H14—C19—H16	109.5
C3—C2—C1	119.52 (15)	C18—C19—H15	109.5
C3—C2—H1	120.2	H14—C19—H15	109.5
C1—C2—H1	120.2	H16—C19—H15	109.5
C2—C3—C4	120.00 (14)	C18—C20—H17	109.5
C2—C3—H2	120.0	C18—C20—H18	109.5
C4—C3—H2	120.0	H17—C20—H18	109.5
C5—C4—C3	119.90 (15)	C18—C20—H19	109.5
C5—C4—H3	120.1	H17—C20—H19	109.5
C3—C4—H3	120.1	H18—C20—H19	109.5
C4—C5—C6	121.52 (15)	C18—C21—H20	109.5
C4—C5—H4	119.2	C18—C21—H22	109.5
C6—C5—H4	119.2	H20—C21—H22	109.5
C1—C6—C5	116.71 (13)	C18—C21—H21	109.5
C1—C6—C7	118.48 (13)	H20—C21—H21	109.5
C5—C6—C7	124.67 (13)	H22—C21—H21	109.5
C8—C7—N1	123.80 (12)	O4—C22—O5	122.69 (13)
C8—C7—C6	118.12 (13)	O4—C22—C16	123.71 (13)
N1—C7—C6	117.96 (12)	O5—C22—C16	113.56 (12)
C7—C8—C9	121.56 (13)	O5—C23—H23	109.5
C7—C8—C14	124.09 (13)	O5—C23—H24	109.5
C9—C8—C14	114.21 (12)	H23—C23—H24	109.5
O2—C9—O1	116.85 (13)	O5—C23—H25	109.5
O2—C9—C8	125.21 (14)	H23—C23—H25	109.5
O1—C9—C8	117.91 (12)	H24—C23—H25	109.5
N1—C10—C11	111.94 (11)	O6—C24—O7	123.96 (13)
N1—C10—H5	109.2	O6—C24—C15	124.46 (13)
C11—C10—H5	109.2	O7—C24—C15	111.57 (12)
N1—C10—H6	109.2	O7—C25—H26	109.5
C11—C10—H6	109.2	O7—C25—H27	109.5
H5—C10—H6	107.9	H26—C25—H27	109.5
C10—C11—S1	111.17 (10)	O7—C25—H28	109.5
C10—C11—H7	109.4	H26—C25—H28	109.5
S1—C11—H7	109.4	H27—C25—H28	109.5
C10—C11—H8	109.4	C2S—C1S—H1SA	109.5
S1—C11—H8	109.4	C2S—C1S—H1SB	109.5
H7—C11—H8	108.0	H1SA—C1S—H1SB	109.5
C13—C12—S1	112.52 (10)	C2S—C1S—H1SC	109.5
C13—C12—H10	109.1	H1SA—C1S—H1SC	109.5
S1—C12—H10	109.1	H1SB—C1S—H1SC	109.5
C13—C12—H9	109.1	O1S—C2S—O2S	117 (2)
S1—C12—H9	109.1	O1S—C2S—C1S	122 (3)
H10—C12—H9	107.8	O2S—C2S—C1S	120.8 (16)
N1—C13—C12	109.89 (11)	O2S—C3S—C4S	104.1 (11)
N1—C13—H11	109.7	O2S—C3S—H3SA	110.9
C12—C13—H11	109.7	C4S—C3S—H3SA	110.9
N1—C13—H12	109.7	O2S—C3S—H3SB	110.9
C12—C13—H12	109.7	C4S—C3S—H3SB	110.9
H11—C13—H12	108.2	H3SA—C3S—H3SB	109.0
C15—C14—O3	109.12 (12)	C3S—C4S—H4SA	109.5
C15—C14—C8	134.25 (13)	C3S—C4S—H4SB	109.5
O3—C14—C8	116.62 (12)	H4SA—C4S—H4SB	109.5
C14—C15—C16	107.78 (12)	C3S—C4S—H4SC	109.5
C14—C15—C24	125.94 (13)	H4SA—C4S—H4SC	109.5
C16—C15—C24	126.25 (12)	H4SB—C4S—H4SC	109.5
C17—C16—C22	121.81 (13)	C7—N1—C13	121.00 (11)
C17—C16—C15	105.20 (12)	C7—N1—C10	120.47 (11)
C22—C16—C15	131.59 (13)	C13—N1—C10	113.69 (11)
N2—C17—O3	119.51 (13)	C17—N2—C18	128.18 (13)
N2—C17—C16	129.71 (13)	C17—N2—H13	115.9
O3—C17—C16	110.72 (12)	C18—N2—H13	115.9
N2—C18—C21	109.92 (13)	C9—O1—C1	121.51 (11)
N2—C18—C20	105.19 (12)	C17—O3—C14	107.11 (11)
C21—C18—C20	109.66 (13)	C22—O5—C23	115.10 (11)
N2—C18—C19	110.86 (12)	C24—O7—C25	114.92 (11)
C21—C18—C19	111.06 (13)	C2S—O2S—C3S	112.1 (10)
C20—C18—C19	109.99 (13)	C12—S1—C11	97.82 (7)
			
O1—C1—C2—C3	−175.67 (13)	C15—C16—C17—O3	2.48 (16)
C6—C1—C2—C3	2.1 (2)	C17—C16—C22—O4	10.8 (2)
C1—C2—C3—C4	1.9 (2)	C15—C16—C22—O4	175.22 (15)
C2—C3—C4—C5	−2.8 (2)	C17—C16—C22—O5	−166.81 (13)
C3—C4—C5—C6	−0.4 (2)	C15—C16—C22—O5	−2.4 (2)
O1—C1—C6—C5	172.56 (13)	C14—C15—C24—O6	58.0 (2)
C2—C1—C6—C5	−5.1 (2)	C16—C15—C24—O6	−119.79 (17)
O1—C1—C6—C7	−3.4 (2)	C14—C15—C24—O7	−123.19 (15)
C2—C1—C6—C7	178.92 (13)	C16—C15—C24—O7	59.05 (18)
C4—C5—C6—C1	4.2 (2)	C8—C7—N1—C13	28.2 (2)
C4—C5—C6—C7	179.92 (14)	C6—C7—N1—C13	−147.75 (13)
C1—C6—C7—C8	0.98 (19)	C8—C7—N1—C10	−125.64 (15)
C5—C6—C7—C8	−174.66 (13)	C6—C7—N1—C10	58.43 (17)
C1—C6—C7—N1	177.15 (12)	C12—C13—N1—C7	139.30 (13)
C5—C6—C7—N1	1.5 (2)	C12—C13—N1—C10	−65.24 (15)
N1—C7—C8—C9	−171.86 (13)	C11—C10—N1—C7	−138.82 (13)
C6—C7—C8—C9	4.1 (2)	C11—C10—N1—C13	65.57 (15)
N1—C7—C8—C14	12.7 (2)	O3—C17—N2—C18	−10.2 (2)
C6—C7—C8—C14	−171.36 (12)	C16—C17—N2—C18	166.87 (15)
C7—C8—C9—O2	175.03 (15)	C21—C18—N2—C17	73.7 (2)
C14—C8—C9—O2	−9.1 (2)	C20—C18—N2—C17	−168.28 (15)
C7—C8—C9—O1	−6.8 (2)	C19—C18—N2—C17	−49.4 (2)
C14—C8—C9—O1	169.05 (12)	O2—C9—O1—C1	−177.30 (13)
N1—C10—C11—S1	−60.96 (14)	C8—C9—O1—C1	4.37 (19)
S1—C12—C13—N1	62.57 (14)	C2—C1—O1—C9	178.43 (13)
C7—C8—C14—C15	61.3 (2)	C6—C1—O1—C9	0.6 (2)
C9—C8—C14—C15	−114.41 (18)	N2—C17—O3—C14	175.06 (13)
C7—C8—C14—O3	−117.28 (15)	C16—C17—O3—C14	−2.50 (16)
C9—C8—C14—O3	66.99 (16)	C15—C14—O3—C17	1.49 (15)
O3—C14—C15—C16	0.03 (16)	C8—C14—O3—C17	−179.57 (12)
C8—C14—C15—C16	−178.64 (15)	O4—C22—O5—C23	−2.8 (2)
O3—C14—C15—C24	−178.07 (12)	C16—C22—O5—C23	174.87 (12)
C8—C14—C15—C24	3.3 (3)	O6—C24—O7—C25	9.0 (2)
C14—C15—C16—C17	−1.50 (16)	C15—C24—O7—C25	−169.88 (12)
C24—C15—C16—C17	176.60 (13)	O1S—C2S—O2S—C3S	9.0 (14)
C14—C15—C16—C22	−167.79 (15)	C1S—C2S—O2S—C3S	−175 (2)
C24—C15—C16—C22	10.3 (2)	C4S—C3S—O2S—C2S	−174.6 (5)
C22—C16—C17—N2	−6.8 (2)	C13—C12—S1—C11	−53.91 (11)
C15—C16—C17—N2	−174.76 (15)	C10—C11—S1—C12	52.18 (11)
C22—C16—C17—O3	170.45 (13)		

### Hydrogen-bond geometry (Å, º)

**Table:**

*D*—H···*A*	*D*—H	H···*A*	*D*···*A*	*D*—H···*A*
N2—H13···O4	0.86	2.25	2.8255 (17)	125
C3—H2···O6^i^	0.93	2.41	3.325 (2)	167
C12—H9···O4^ii^	0.97	2.44	3.1531 (18)	130
C12—H10···O1*S*^iii^	0.97	2.48	3.208 (5)	132
C19—H15···O3	0.96	2.42	3.0090 (19)	119
C23—H23···S1^iv^	0.96	2.78	3.5639 (17)	139
C25—H26···S1^v^	0.96	2.83	3.7406 (18)	159

Symmetry codes: (i) −*x*+2, −*y*, −*z*+1; (ii) −*x*+1, −*y*, −*z*; (iii) *x*, *y*, *z*−1; (iv) −*x*+1, *y*+1/2, −*z*+1/2; (v) *x*, −*y*−1/2, *z*+1/2.
