# Supplementary figures and images for: Crystal structure of 3,4-dimethyl 2-(tert-butyl­amino)-5-[2-oxo-4-(thio­morpholin-4-yl)-2H-chromen-3-yl]furan-3,4-di­carboxyl­ate ethyl acetate hemisolvate
Source: Acta Crystallogr E Crystallogr Commun. 2015 Nov 28;71(Pt 12):o1003–4. doi: 10.1107/S2056989015021970 (PMC4719946; doi:10.1107/S2056989015021970)

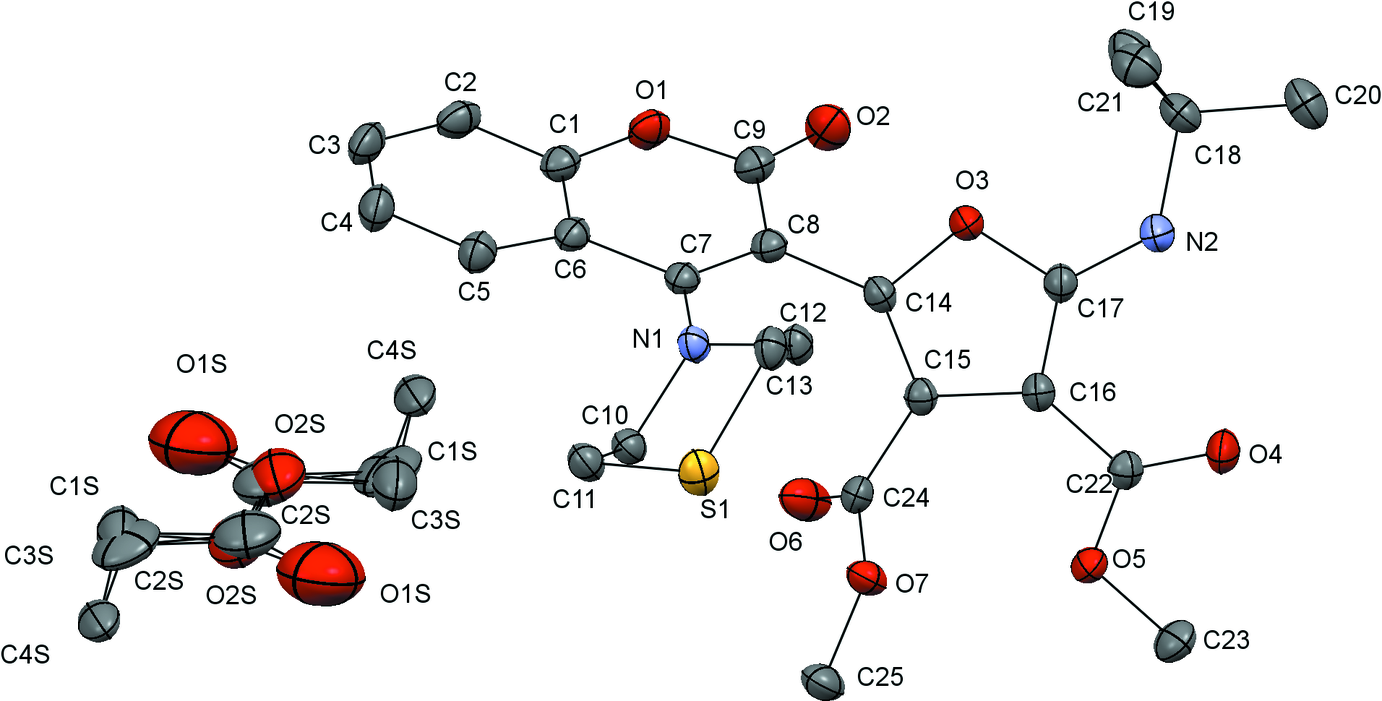

Supplement: Supplementary file 4 [file e-71-o1003-fig1.tif]

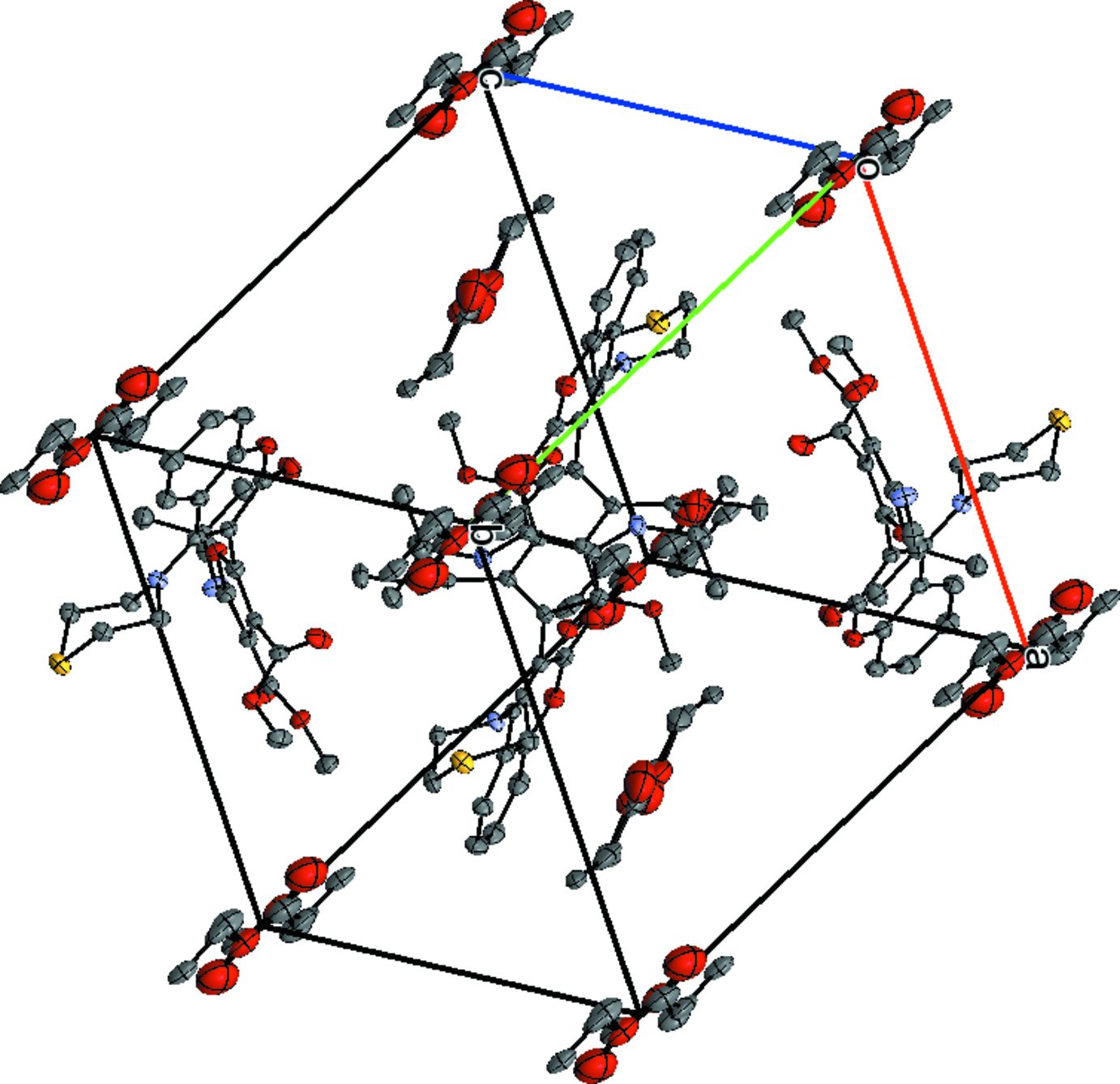

Supplement: Supplementary file 5 [file e-71-o1003-fig2.tif]
